# New In Situ Amphipathic Polymerization-Modified Titanium Quantum Dots: Application as a High-Performance Water-Lock-Breaking Agent in Tight Gas Reservoirs

**DOI:** 10.3390/molecules31081338

**Published:** 2026-04-19

**Authors:** Haibo Li, Hongxing Xu, Lei Yue, Yining Zhou, Yanhong Li, Kongjie Wang, Changzhou Tao, Boli Yang, Long Chai, Haihong Feng

**Affiliations:** Changqing Downhole Technology Company, CNPC Chuanqing Drilling Engineering Company Limited, Xi’an 710018, China

**Keywords:** titanium quantum dot, water lock damage, decreasing surface tension, wettability alteration, tight gas reservoir

## Abstract

In this paper, to remove the water lock effect in tight gas reservoirs, amphipathic polymer-modified titanium quantum dots (PTQs) were synthesized via in situ polymerization, showing a hyper-branched structure and an excellent synergistic effect with the nonionic fluorocarbon surfactant to break the water lock. The molecular structure, fluorescent property, and micromorphology of the PTQs were obtained. The surface activity and wettability alteration of rock are discussed. Results show that PTQs have zwitterionic hydrophilic groups and the hydrophobic structure of long-chain groups on their molecular structure. PTQ fluid, with a median particle size of 3.6 nm, showed strong green fluorescence and had excellent dispersibility in 50,000 mg/L of standard saline fluid at 120 °C. Additionally, the surface tension decreased to 18.6 mN/m at a PTQ concentration of 0.08%. At a 0.1% concentration, PTQ fluid altered the water wettability of tight sandstone to 67.2°, which resulted in lower capillary resistance. Furthermore, the surfactant (PHPE) had a good synergistic effect with the PTQs to decrease surface tension and alter the wettability of the sandstone surface, leading to lower surface tension and significant amphiphobicity. The strong surface activity of PTQs results from their specific molecular structure, which enables electrostatic attraction, quantum size effects, hydrogen bonding, and van der Waals forces between the inter-polar molecules of PTQs and the surface of sandstone to forcefully eliminate the water lock effect. This study offers key guidance for the development of a high-performance water-lock-breaking agent and application of titanium quantum dots in tight gas reservoirs.

## 1. Introduction

Capillary resistance is a key factor causing the water lock effect in the later stages of gas wells, which results in serious reservoir damage that inhibits the flow and output of natural gas, especially in tight gas reservoirs [1,2,3]. The formula for capillary resistance (Pc) is shown in Equation (1).

(1) $$P_{c}=\frac{2\sigma cos\;\theta }{r}$$

where $P_{c}$ is the capillary resistance in Pa; σ is the surface tension in mN/m; θ is the contact angle in °; and r is the capillary tube radius in m.

The key methods for breaking the water lock effect are decreasing surface tension, altering wettability toward hydrophobicity, and enlarging the radius of the pore-throat. Research into these three areas has been done for many years, especially for decreasing surface tension and altering wettability toward hydrophobicity. Wang Liang et al.’s results showed that when reservoir wettability was changed from hydrophilic to hydrophobic, correspondingly, capillary forces acted as a driving force that promoted gas flow out [4]. SALEHI M et al. found that ion pair formation between the charged head groups of surfactant molecules and the adsorbed crude oil components on the rock surface were more effective than the hydrophobic interactions between them in changing the rock’s wettability toward a more water-wet state [5]. LI Y F et al. synthesized a novel fluoropolymer gas-wetting alteration agent. The test yield (84.36%) was close to the predicted yield (83.66%). Furthermore, the contact angle of water and n-hexadecane on the core surface increased from 34° and 0° to 126° and 102° after being treated with 1.5 wt.% fluoropolymer solution. The surface free energy was reduced from 73 mN/m to 8.2 mN/m at equal mass fractions [6]. AMINNAJI M et al. studied changes in the wettability of carbonate and sandstone rocks toward intermediate gas wetting using a nanofluid. The results showed that the nanofluid used in their work considerably changed the wettability of both surfaces for preferential gas wetting. Initial oil saturation reduced the impact of the nanofluid on wettability change [7]. SAFAEI A et al. synthesized Fe_3_O_4_ nanoparticles coated with two polymers, poly (vinyl alcohol) (PVA) and hydroxyapatite (HAP). The results showed that the imbibition rate of water and condensate oil noticeably reduced after the wettability alteration by core–shell nanostructures. The pressure drop and breakthrough time also significantly diminished. Compared to the iron oxide NPs coated with PVA, those coated with HAP significantly improved the wettability of the rock samples from oil wetting to gas wetting [8]. WANG Y L et al. synthesized a gas-wetting alteration agent, N, N-bis(perfluorooctyl)imine acetate sodium (BFAS). The results showed that after treatment with 0.5 wt.% BFAS, the contact angles of water and n-hexadecane on the surface of the rock increased from 36° and 0° to 140° and 119°, respectively. The surface free energy rapidly reduced from primeval 72 mN/m to 3.4 mN/m after treatment with 0.3 wt.% BFAS [9]. Karandish G et al. developed a new chemical treatment to alter the wettability of carbonate rocks, using an anionic fluoro-surfactant mixture and a solvent. The contact angles were increased and the water/oil imbibition were reduced significantly after use of the treatments. In addition, the treatment enhanced the gas relative permeability by a factor of 1.7 [10]. Saboori R A et al. investigated the effect of fluorine-doped silica coated with fluorosilane nanofluid on the wettability alteration of carbonate rock. The results showed that the wettability was altered from strongly oil-, condensate-, and water-wet to strongly gas-wet. The contact angles for water, condensate, and crude oil were altered from 37.95°, 0°, and 0° to 146.47°, 145.59°, and 138.24°, respectively. Meanwhile, the water, condensate, and oil imbibition decreased by more than 87, 88, and 80% [11]. Ni X et al. synthesized amphiphobic nanofluids by combining nano-silica, multiwalled carbon nanotube (MCNTs) and trichloro (1H,1H,2H,2H-perfluorooctyl) silane (PFOS) to address the water-blocking and water-sensitivity issues in low-permeability sandstone reservoirs. These fluids possessed bead chain structures with a special chemical property that altered the sandstone core surface from amphiphilic to amphiphobic [12]. Naghizadeh A et al. investigated the influence of a silica nanofluid modified by fluorine groups on the wettability alteration of carbonate. The wettability of core samples changed from liquid-wet to gas-wet due to the surface treatment, and a higher contact angle was achieved for carbonate cores with less dolomite than calcite [13].

Surfactants or active polymers are usually adopted for the development of water-lock-breaking agents, especially fluorocarbon chemicals, which have excellent performance in altering rock wettability toward amphiphobicity. In addition, modified nanomaterials have also been used in this research. However, amphipathic polymer grafting onto nanoparticles to form a hyper-branched structure and enable high surface activity via in situ polymerization has rarely been reported. Furthermore, compared with traditional nanoparticles, quantum dots have an inherent size advantage that helps avoid potential blocking damage in tight reservoirs and provides a significantly stronger size effect, enhancing surface activity [14,15]. Meanwhile, reports about the synthesis of amphipathic polymer-modified quantum dots and their application for breaking the water lock effect have not been found. Therefore, in this paper, amphipathic polymer-modified titanium quantum dots (PTQs) were synthesized via in situ polymerization, and their synergistic effect with a fluorocarbon surfactant in the preparation of a water-lock-breaking agent for tight gas reservoirs was also studied. Their molecular structure, fluorescent property, and micromorphology were observed. Moreover, the surface activity performance and wettability alteration of rock are also discussed. 

## 2. Results

### 2.1. Characterization of PTQs

**Figure 1:**
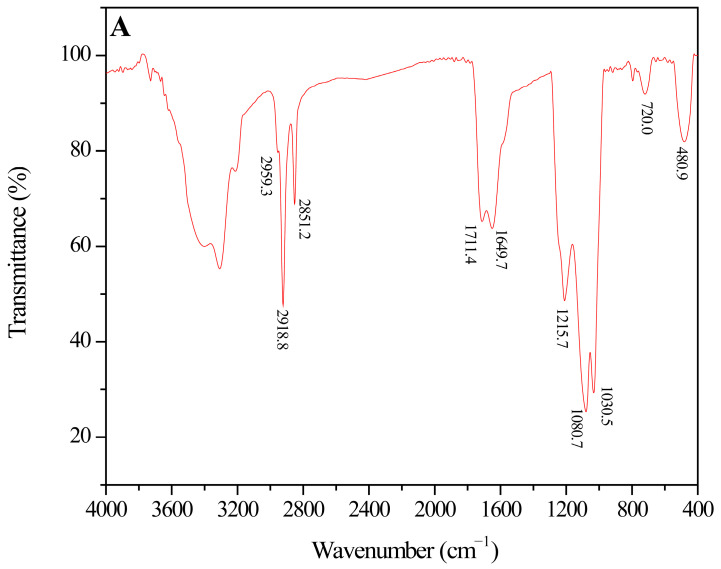

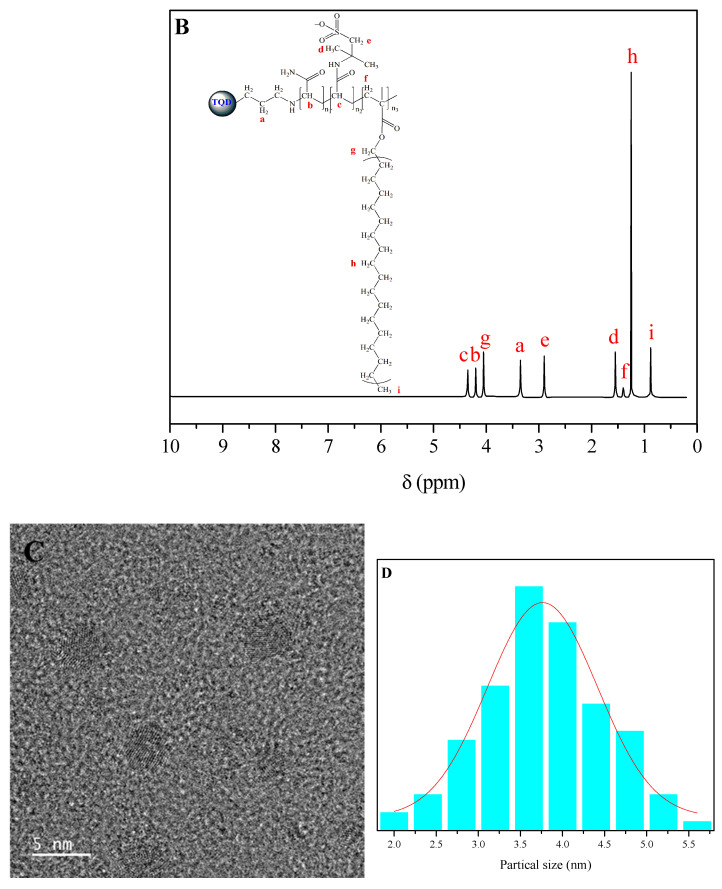

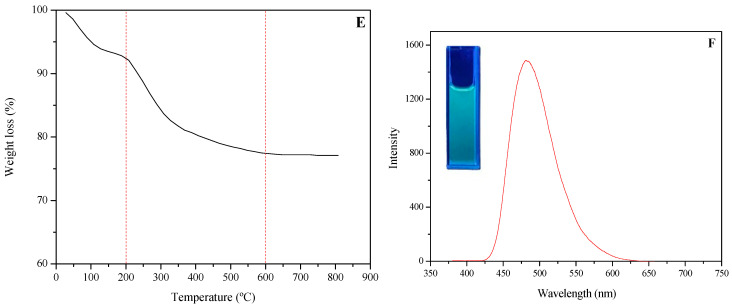
Micro-characteristics of PTQs: infrared spectroscopy (FT-IR) of PTQs (**A**); ^1^H NMR spectrum of PTQs dispersed in D_2_O at 400 MHz (**B**); micromorphology of PTQs from HRTEM (**C**); particle size distribution of PTQs obtained by Image J 1.8.0 (**D**); TG of PTQs under a N_2_ atmosphere (**E**); fluorescence fingerprint of PTQs nanofluid under UV light (**F**).

Capillary resistance plays a crucial role in the generation of water lock in tight gas reservoirs. Therefore, decreasing surface tension and altering wettability are the optimal methods to eliminate capillary resistance and break the water-lock effect. With a molecular structure similar to that of surfactants, amphiphilic polymer-grafted titanium quantum dots were synthesized as a water-lock-breaking agent. The micro-characteristics of the PTQs are shown in Figure 1.

The results in Figure 1A show that the peak at 480.9 cm^−1^ is the stretching vibration of Ti–O–Ti, which indicates that titanium quantum dots were synthesized. The peak at 1030.5 cm^−1^ corresponds to the vibration adsorption peak of Ti–O–Si, which indicates that APTES was grafted onto the surface of the TQDs. The peak at 720.0 cm^−1^ corresponds to the rocking vibration of —CH_2_— in long carbon chains, while the peaks at 2851.2 cm^−1^ and 2918.8 cm^−1^ correspond to the symmetrical stretching vibration and asymmetric stretching vibration of —CH_2_—, respectively. The peak at 1711.4 cm^−1^ corresponds to the stretching vibration peak of C=O in SMA. The peak at 2959.3 cm^−1^ corresponds to the stretching vibration peak of —CH_3_, while the peak at 1215.7 cm^−1^ corresponds to the asymmetric stretching vibration of S=O in —SO_3_H in AMPS. The peak at 1649.7 cm^−1^ corresponds to the stretching vibration peak of C=O in AM. Finally, the broad peak at 3300~3400 cm^−1^ corresponds to the vibration peaks of O—H and N—H. This indicates that the AM-SMA-AMPS co-polymer was grafted onto the surface of TQDs via in situ polymerization, forming an amphiphilic structure and providing strong surface activity.

In Figure 1B, the multiple peaks at δ 4.35 ppm correspond to the main-chain —CH linked to AMPS. The multiple peaks at δ 4.20 ppm correspond to 1H in the main-chain —CH linked to AM. The triplet peaks at δ 4.05 ppm correspond to 2H of O—CH_2_— in the ester group in SMA. The triplet peaks at δ 3.35 ppm correspond to 2H of CH_2_—NH linked to TQDs. The chemical shift at 2.90 ppm correspond to the triplet peaks of 2H in the CH_2_—SO_3^−^_ of AMPS, resulting from the deshielding effect. The chemical shift at 1.55 ppm corresponds to the single peak of 6H in the —C(CH_3_)_2_ of AMPS. The multiple peaks at δ 1.41 ppm correspond to the 2H of the —CH_2_ adjacent to the ester group in SMA. The clear single peak at δ 1.25 ppm and the triplet peaks at δ 0.88 ppm correspond to the 2H of —(CH_2_)_n_— and the 3H of the terminal —CH_3_ in the long alkyl chain in SMA, respectively, which mainly results from the electron-shielding effect. The results are in good agreement with the infrared analysis, indicating that the product was successfully synthesized.

It can be seen in Figure 1C that titanium quantum dots were generated and their shape was mainly spherical and semispherical. An excellent dispersion state was also acquired. The results in Figure 1D indicate that their size was smaller than 10nm, with a median size of about 3.6nm, which is consistent with the essential characteristics of quantum dot nanomaterials [16]. Because of this, PTQs can freely migrate in tight gas reservoirs and absorb onto the surface of sandstone to decrease surface tension and change wettability without causing any formation damage.

The results in Figure 1E show that the weight decrease in the PTQ sample can be divided into three stages, including a slow decline from 30 °C to 200 °C, a quick decrease from 200 °C to 600 °C, and a steady phase from 600 °C to 800 °C. The weight loss in the first stage was mainly due to the evaporation of constitutional water and adsorbed water on the PTQ surface. The weight loss in the second stage was caused by pyrolysis of the polymer grafted onto the PTQ surface. However, more than 75% of non-pyrolyzable residuals remained above 800 °C. The non-pyrolyzable residue consisted of TiO_2_ nanoparticle crystals with a pyrolysis temperature of 2000 °C. The above-obtained results are strong evidence for the formation of titanium quantum dots and indicate that the amphiphilic polymer was grafted onto the PTQ surface. Then, the grafting rate was calculated as about 15.0%. According to the amount of each reactant added during the synthesis of the PTQs and the final grafting rate of the co-polymer on the surface of the TQDs, the estimated polymer conversion rate was nearly 90%.

In Figure 1F, it can be seen that green fluorescence was clearly exhibited by the PTQ nanofluid under ultraviolet light, and the fluorescence spectrum shows a characteristic peak at 490 nm upon excitation of 380 nm, which indirectly confirms the quantum dot feature of the PTQs.

### 2.2. Surface Tension of PTQ Nanofluid

**Figure 2:**
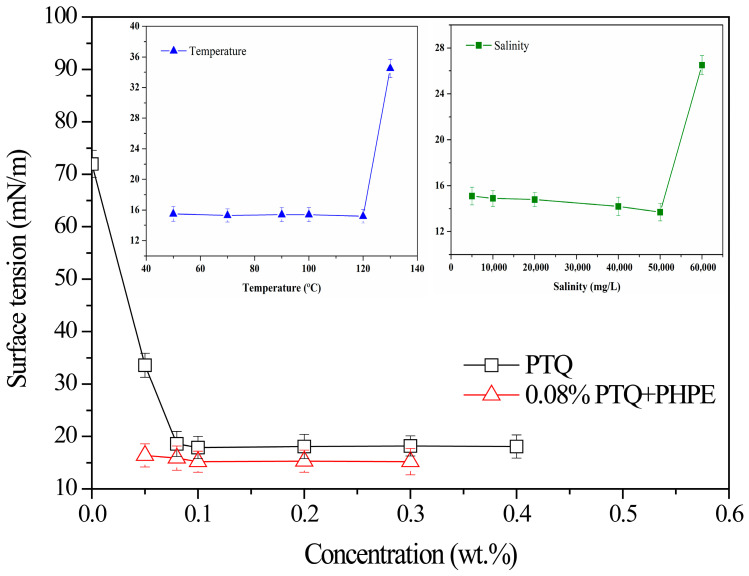
The surface tension of PTQ nanofluid versus concentration under the influence of salinity and temperature.

Decreasing the surface tension of water is a powerful method for the elimination of water lock. Therefore, the surface tension of PTQ nanofluid was studied under the influence of salinity and temperature. Furthermore, the synergistic effect between PTQs and a fluorocarbon surfactant (PHPE) in decreasing surface tension is discussed. Before testing, the PTQ nanofluid was placed in the experimental salinity and temperature to age for 24 h. The salinity was controlled by the concentration of KCl. The obtained results are shown in Figure 2.

It was found that the surface tension significantly decreased as the PTQ concentration and PHPE concentration increased. The surface tension reached 18.6 mN/m at 0.08 wt.% PTQ concentration. This can be considered as the critical micelle concentration of PTQs, which is significantly lower than the conventional surfactant [17,18]. Moreover, due to the effect of PHPE, the surface tension of 0.08% PTQ was further decreased. The surface tension reduced to 15.2 mN/m when the PHPE concentration was 0.1 wt.% and then remained in a steady state. There was an excellent synergistic effect between the PTQs and the fluorocarbon surfactant. In addition, the salinity and temperature had a significant influence on the surface tension of the PTQ nanofluid. The surface tension of 0.08% PTQ + 0.1% PHPE fluid decreased as the salinity increased. When the salinity was 50,000 mg/L, the surface tension was 13.7 mN/m, and when the salinity was further increased, the surface tension suddenly increased, mainly due to the compressed double-electric layer. Therefore, the salinity resistance for this system is 50,000 mg/L. Interestingly, the surface tension of 0.08% PTQ + 0.1% PHPE fluid was steady when the temperature was lower than or equal to 120 °C but significantly increased when the temperature was further increased. The temperature tolerance for this system is 120 °C, which indicates excellent thermostability.

### 2.3. Wettability Alteration

#### 2.3.1. Contact Angle of Sand Slice Under Effect of PTQs

**Figure 3:**
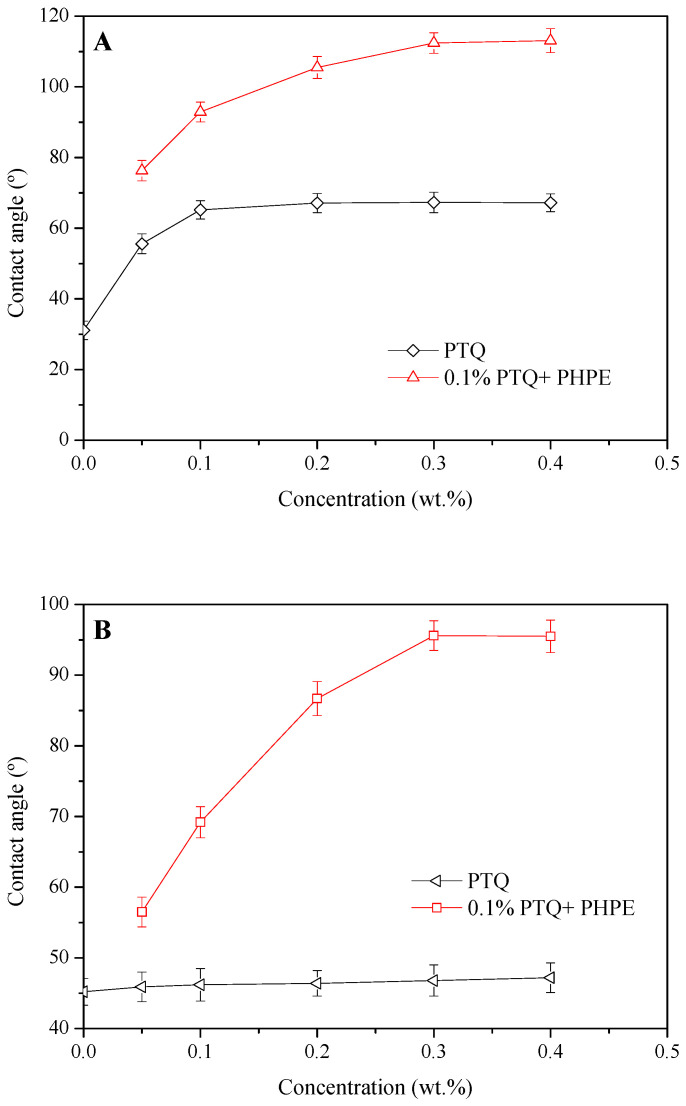
The water/oil contact angle ((**A**), deionized water; (**B**), hexadecane) of sandstone slice under the effect of PTQs.

Gas wettability alteration, as a crucial parameter of water-lock-breaking agents, helps decrease capillary resistance and increase gas permeability. The water/oil contact angle of a sandstone slice under the effect of PTQs is shown in Figure 3.

It was found that the initial water contact angle and the initial oil contact angle of the sandstone slice were 31.1° and 45.2°, respectively, indicating preferential hydrophilicity and hydrophobicity. Under the effect of PTQs, the water contact angle first increased and then became steady as the PTQ concentration increased. When the PTQ concentration was 0.1 wt.%, the final stable contact angle was 67.2°, indicating increased hydrophobicity. However, there has no obvious change in the oil contact angle of the sandstone rock after PTQ treatment. In addition, when PHPE was mixed with 0.1% PTQ, the hydrophobicity and lipophobicity on the surface of the sandstone were all further increased. When the PHPE concentration was greater than 0.3%, the water contact angle reached 112.4° and the oil contact angle was 95.6°. Then, they entered a stable phase. Under the influence of the used fluorocarbon surfactant, the hydrophobicity alteration induced by the PTQ nanofluid was significantly enhanced and strong lipophobicity of the sandstone surface was also obtained, mainly due to the existence of the C—F bond. Therefore, the gas wettability of the sandstone surface was distinctly improved. 

#### 2.3.2. Surface Free Energy of Sandstone Under Influence of PTQs

**Figure 4:**
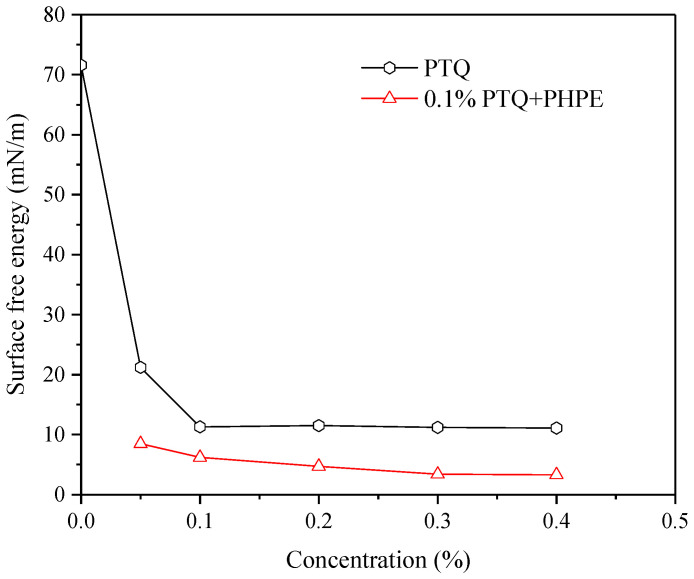
The surface free energy of sandstone under the influence of PTQs.

Surface free energy was employed to reflect the wettability alteration in the sandstone surface under the effect of PTQs. The surface free energy of the sandstone under the effect of PTQs is shown in Figure 4.

The results show that the surface energy of the sandstone was significantly decreased and then became stable as the PTQ concentration increased. It decreased from 71.6 mN/m to 11.3 mN/m when the PTQ concentration was 0.1%. Subsequently, the surface free energy of the sandstone further declined when PHPE was added to the PTQ nanofluid, and it reaching 3.4 mN/m when the PHPE concentration reached 0.3%. As the PHPE concentration continued to increase, the surface free energy showed no obvious change. These results indicate that the dispersion force and the polar force were weakened by the interaction between PTQ and PHPE, corresponding to the enhanced amphiphobicity on the surface of the sandstone.

#### 2.3.3. Liquid Level

**Figure 5:**
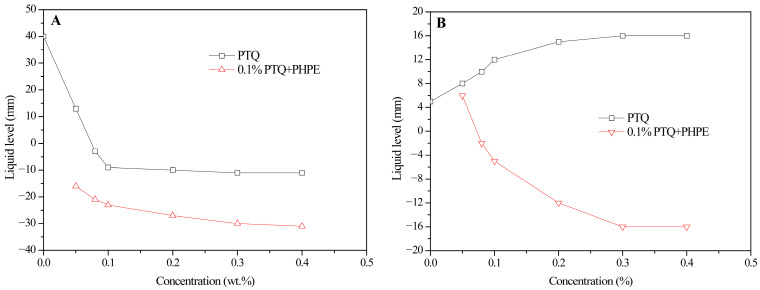
The liquid levels of deionized water (**A**) and hexadecane (**B**) in a glass capillary tube under the influence of PTQs.

A glass capillary tube was also adopted to characterize the wettability alteration induced by PTQs. The liquid levels of deionized water and hexadecane in the glass capillary tube under the influence of PTQs are shown in Figure 5.

As shown in Figure 5, the liquid level of deionized water significantly decreased with the increase in PTQ concentration, but that of hexadecane significantly increased. PTQs had a significant influence on the water-wetting behavior of the capillary tube, inhibiting the increase in the liquid level of deionized water. Therefore, an enhanced oil-wetting behavior was induced, increasing the liquid level of hexadecane. However, when the PHPE was added to the 0.1% PTQ dispersing fluid, the amphiphobicity of the glass capillary tube improved significantly. The liquid level of water further decreased, while the liquid level of hexadecane also decreased from +15 mm to −16 mm. It can be seen that under the combined effect of PTQs and PHPE, the amphiphobicity of the capillary tube was significantly enhanced, which agrees with the results from the contact angle test.

#### 2.3.4. Spontaneous Imbibition

**Figure 6:**
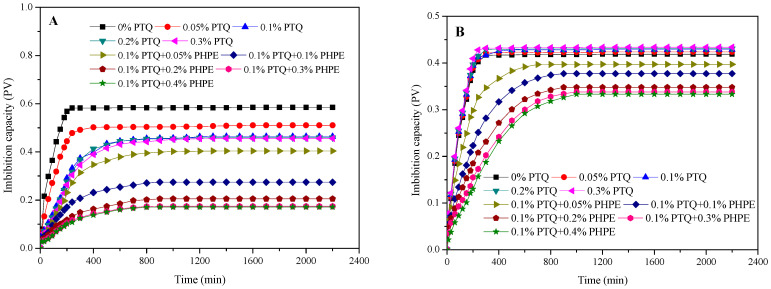
The imbibition volume of deionized water (**A**) and hexadecane (**B**) under the influence of PTQs and PHPE.

Spontaneous imbibition of sandstone was adopted to evaluate the wettability alteration under the effect of PTQs and PHPE. The imbibition capacity of the liquid used under the influence of PTQs and PHPE is shown in Figure 6.

Before treatment with PTQs and PHPE, the imbibition volumes of deionized water and hexadecane rapidly increased and then entered a stable state over time, with equilibration times of 200 min and 270 min, respectively. Meanwhile, the saturated imbibition volumes of deionized water and hexadecane were 0.585 PV and 0.426 PV, respectively. However, after treatment with PTQs, the imbibition volume and imbibition rate of deionized water were significantly inhibited. The imbibition volume decreased to 0.463 PV when the PTQ concentration was 0.1% and became steady when the PTQ concentration was further increased. The imbibition capacity of hexadecane exhibited no clear variation under the effect of PTQs. Moreover, after treatment with 0.1% PTQ and PHPE, the imbibition capacity of hexadecane and deionized water were all weakened by the synergistic effect between them. This interaction was more powerful as the PHPE concentration increased until stabilizing when the PHPE concentration exceeded 0.3%. The imbibition volumes of hexadecane and deionized water declined to 0.337 PV and 0.174 PV at a 0.3% PHPE concentration. At the same time, the equilibration times for hexadecane and deionized water decreased to 840 min and 520 min, respectively. These results are in line with those of the contact angle measurements.

## 3. Discussions

**Figure 7:**
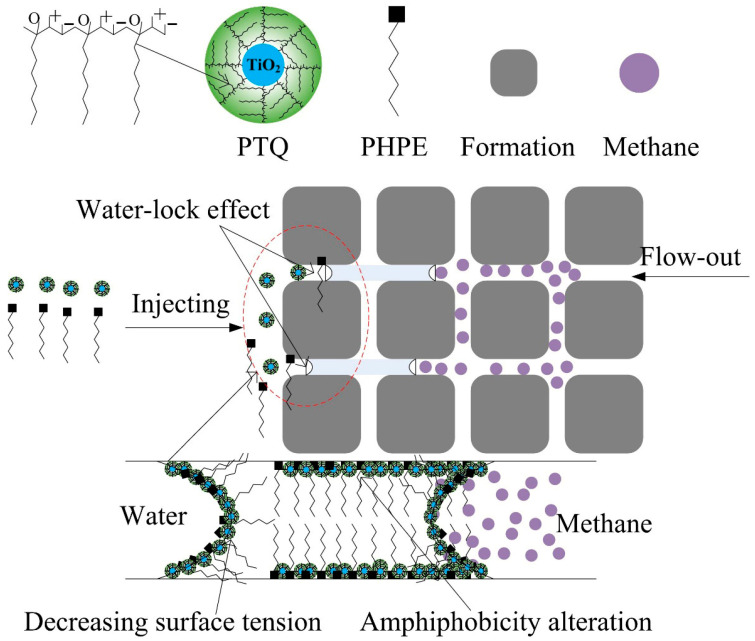
Schematic diagram of the interaction mechanism of PTQs for breaking water lock.

The water lock effect plays a vital role in the production of methane in the tight sandstone of the Shanxi Formation in the Sulige gas field. Its key mechanism is that the existence of capillary resistance decreases gas permeability and significantly inhibits the discharge of methane. The key solutions for eliminating capillary resistance include decreasing surface tension, altering wettability, and enlarging the pore-throat radius, with PTQs mainly addressing the first two. A schematic diagram of the interaction mechanism of PTQs for breaking water lock is shown in Figure 7.

Amphiphilic polymer-grafted titanium quantum dots contain a large number of strongly lyophobic groups with long hydrocarbon chains, as well as many hydrophilic groups with anionic–cationic groups on its surface, similar to a hyper-branched polymer assembled from zwitterionic surfactants.

Therefore, PTQs can spontaneously arrange on the interface of water to significantly reduce surface free energy, with the long hydrocarbon chains distributed in the gas phase and the anionic–cationic groups remaining in the water phase. Under the effect of electrostatic attraction and the quantum size effect, the gap between two PTQs and two active molecules is significantly decreased. This significantly improves the interface density of PTQs [19,20]. Consequently, these intermolecular actions of PTQs cause a higher decrease in surface tension than other conventional active chemicals [21]. Meanwhile, the nonionic fluorocarbon surfactant PHPE exhibits powerful surface activity and decreases surface free energy. Its polar hydrophilic groups form stronger hydrogen bonds with the polar groups on the surface of PTQs than conventional hydrocarbon, leading to an excellent synergistic effect on the gas–water interface to further decrease the surface tension and break water lock [22].

**Figure 8:**
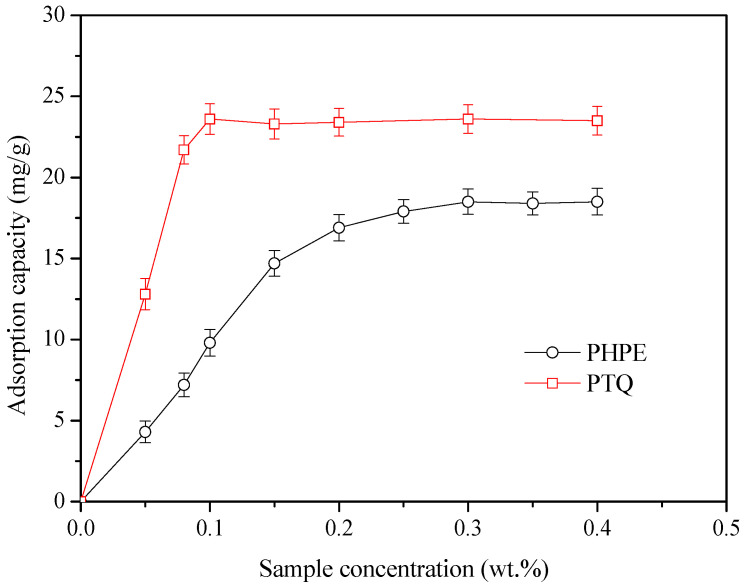
The adsorption capacity of PTQs on sandstone surface.

Due to the existence of polar groups on the surfaces of PTQs and PHPE, strong hydrogen bonding, electrostatic effects, and van der Waals forces occur between PTQs and the sandstone surface [23,24,25]. Therefore, the strong adsorption of PTQs on the sandstone surface alters its wettability. The adsorption capacities of PTQs and PHPE are shown in Figure 8.

It was found that the adsorption capacity of PTQs first increased and then stabilized as the used concentration increased. The stable stage is also called the equilibrium adsorption concentration. Under this condition, the adsorption and desorption behaviors of the active chemicals are in a state of dynamic equilibrium. PTQs exhibited a higher adsorption capacity of 23.6 mg/g and a lower equilibrium adsorption concentration of 0.1% on the surface of sandstone compared to PHPE, which indicates that there was a strong intermolecular force between them [26]. Hence, the hydrophilic parts of PTQ and PHPE spontaneously absorb onto the surface of sandstone, while the long hydrocarbon chain and fluorocarbon chain extend into the gas phase, which results in significant wettability alteration of the sandstone surface. Therefore, in addition to significantly decreasing surface tension, amphiphobicity is an essential condition for injected active chemicals to effectively break the water lock effect. The amphiphobicity of sandstone is also significantly enhanced under the combined effects of PHPE and PQE [27,28]. Meanwhile, extra osmotic pressure is generated, which promotes the desorption of methane [29,30]. 

## 4. Materials

Tetra isopropyl titanate (TIPT, 99.99%), acetic acid (CH_3_COOH, 99.99%), stearyl methylacrylate (SMA, 99.99%), acrylamide (AM, 99.99%), 2-Acrylamide-2-methylpro panesulfonic acid (AMPS, 99.99%), perfluorohexyl ethanol polyoxyethylene ether (PHPE, 99%), and (3-amino propyl) triethoxy silane (APTES, 99%) were all purchased from Aladdin (Shanghai, China). Absolute ethanol (C_2_H_5_OH, 99%), polyethylene glycol 2000 (PEG, 99%), ammonium hydroxide (25~28% in water), ethyl acetate (EA, 99%), Tween 60 (T-60, 99%), potassium peroxodisulfate (99%), potassium chloride (KCl, 99%), and sodium hydroxide (NaOH, 99%), n-hexadecane (99%) were all purchased from CHRON CHEMICALS (Chengdu, China). All chemicals received were not further treated. Deionized water was self-made in the laboratory by the HHitech Smart-S water purification system (conductivity <10^−1^ μS/cm). Natural sandstone cores were obtained from the Shanxi Formation in the Sulige gas field. The basic properties of the sandstone cores are shown in Table 1.

## 5. Methods

### 5.1. Synthesis of PTQ

First, 8 mL of TIPT was added to 4 mL of acetic acid at a volume concentration of 25%. Then, absolute ethanol and 1 g of PEG were added to this mixed solution until the total volume was 100 mL to prepare the titanium precursor solution. Then, the precursor solution was placed in a high-temperature and high-pressure reactor and reacted at 150 °C for 3 h. Finally, the titanium quantum dots (TQDs) were collected by a 3 kDa dialysis bag, washed with deionized water, and dried at 50 °C for 36 h.

Next, 2 g of dried TQDs was dispersed in 90 mL of ethanol fluid at a volume concentration of 80%. Then, 20% of ammonium hydroxide fluid was used to adjust the pH of the quantum dot fluid to about 10. Then, 10 mL of ethanol fluid containing 0.3 g of APTES was added dropwise to the above mixed fluid, and the reaction was carried out at 55 °C for 14 h. Thereafter, the product was collected by a 3 kDa dialysis bag, washed by deionized water, and dried in a vacuum oven at 50 °C for 36 h. Thus, amino-modified titanium quantum dots (ATQDs) were obtained.

**Figure 9:**
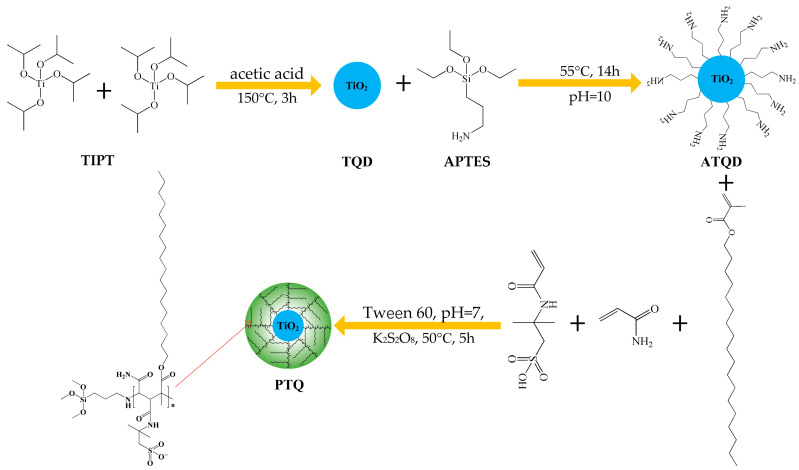
The synthetic route of PTQs.

Subsequently, 1 g of dried ATQDs and 1 g of Tween 60 were dispersed and dissolved in 40 g of deionized water. Then, 0.05 g of SMA, 0.1 g of AM, and 0.05 g of AMPS were added to the above-mentioned solution, and deionized water was added until the total weight of fluid was 100 g. Then, 2% of NaOH fluid was added to this mixed solution until the pH was equal to 7. Thereafter, 2 ml of fluid containing 0.04 g of potassium peroxodisulfate was added dropwise to the above mixed solution. The reaction was done at 50 °C for 5 h. Finally, the produced PTQs were extracted by ethyl acetate, separated by a high-speed centrifuge, washed by deionized water, and dried at 60 °C for 36 h. The synthetic route of PTQs is shown in Figure 9.

### 5.2. Characterization

A Fourier transform infrared spectrometer (FT-IR, Nicolet 5700, Waltham, MA, USA) was employed to analyze the infrared spectroscopy of the PTQs within the wavenumber range of 4000–400 cm^−1^ at room temperature. The sample was a laminate prepared by pressing the mixture of dried KBr and PNS at a ratio of 100:1. The PTQs were homogeneously dispersed into heavy water (D_2_O), and an HNMR (JEOL/JNM-ECZ400S/L1, Tokyo, Japan) was utilized to further analyze the molecular structure of PTQ, operating at 400 MHz for H. A transmission electron microscope (TEM, JEOL JEM-2100, Tokyo, Japan) was used to observe the morphological features of the PTQs to analyze their dispersion state and grain size distribution. A thermal gravimetric analyzer (TGA, Mettler Toledo TGA2, Zurich, Switzerland) was adopted to evaluate the thermal stability of the compound and the surface grafting rate of the PTQs at 40 to 800 °C and at a 10 K/min heating rate. The grafting rate of polymers on the surface of the TQDs is defined as the differential value between the weight loss rate of the TQDs and PTQs. The Darkroom UV analyzer and a spectrofluorophotometer (LS55, PerKinEImer, Waltham, MA, USA) were used to observe the fluorescence fingerprint of the PTQ fluid.

### 5.3. Surface Tension

The surface tension of the samples was measured via the pendant drop method using an interfacial tensiometer (DSA100, KRÜSS, Hamburg, Germany) at room temperature. The prepared sample was placed in the measuring cell, and a syringe with a bent needle was used to inject a spindle-shaped pendant bubble. Then, the surface tension was calculated by matching the shape of bubble. All experiments were carried out three times, and the final value was taken as the average of these three values. All data are presented as the mean value ± standard deviation (SD).

### 5.4. Adsorption of PTQs on the Sandstone Surface

Natural sandstone core samples were crushed into fine particles, and the crushed sandstone was sieved to obtain 100–200 mesh powder. Subsequently, the sandstone powder was subjected to Soxhlet extraction with a toluene–ethanol mixture (1:1, *v*/*v*) for 24 h to completely remove residual crude oil and organic impurities. After extraction, the powder was rinsed with petroleum ether to eliminate residual solvent, then dried in a vacuum drying oven at 105 °C to a constant weight, and stored in a desiccator for subsequent use to avoid moisture absorption and contamination. The ultraviolet-visible spectrophotometer (UV-2600, SHIMADZU, Kyoto, Japan) was employed to evaluate the adsorption capacity of PTQs on the surface of sandstone at room temperature. The calibration curve of the PTQ concentration was done first to calculate the adsorption capacity of the PTQs, and the oscillation equilibrium method was used to determine the adsorption equilibrium of the PTQs on the surface of sandstone. The formula for the static adsorption capacity is shown in the following Equation (2):

(2) $$\Gamma =\frac{V(c_{0}\;-\;c_{t})}{m}$$

where Γ is the static adsorption capacity, mg/g; V is the volume of PTQ nanofluid, mL; $c_{0}$ is the initial concentration of PTQ nanofluid, mg/L; $c_{t}$ is the final concentration of PTQ nanofluid after adsorption balance, mg/L; m is the weight of sandstone, g. All experiments were carried out three times, and the average of these three values was taken as the final value. All data are presented as the mean value ± standard deviation (SD).

### 5.5. Contact Angle 

The sandstone sample was cut by a linear cutting machine (STX-1203, KEJING, Shenyang, China) to sandstone slices 50 mm in diameter and 5 mm in height. The polished sandstone slice was treated by immersion and aged at 120 °C for 24 h. Then, the aged sandstone slice was taken out and dried at 55 °C for 12 h. The sessile drop method using an interfacial tensiometer (DSA100, KRÜSS, Hamburg, Germany) was used to measure the contact angle of deionized water or n-hexadecane on the original and treated rock surfaces. A drop of deionized water or n-hexadecane was placed on the surface of the original or treated rock by a syringe with a needle. Then, the morphology of the sessile droplet on the rock surface was matched using the tangent equation, and the contact angle was calculated by the software’s goniometry tool. All experiments were carried out three times, and the final value was taken as the average of these three values. All data are presented as the mean value ± standard deviation (SD). 

### 5.6. Surface Free Energy

The Owens two-liquid method was adopted to obtain the surface free energy, which is also a key factor for characterizing the wettability of a rock surface. The calculation equation for it is as follows:

(3) $$\gamma_{s}\;=\;\gamma_{s}^{D}\;+\;\gamma_{s}^{P}$$

(4) $$\gamma_{L}\left(1+cos\;\theta \right)=2{\left(\gamma_{s}^{D}\gamma_{L}^{D}\right)}^{1/2}+2{\left(\gamma_{s}^{P}\gamma_{L}^{P}\right)}^{1/2}$$

where $\gamma_{s}$ is the surface free energy of the solid, mN/m; $\gamma_{s}^{D}$ and $\gamma_{s}^{P}$ are the dispersion force and polar force of the solid, respectively, mN/m; $\gamma_{L}$ is the surface free energy of liquid, mN/m; θ is the contact angle of the liquid on the solid, °; $\gamma_{L}^{D}$ and $\gamma_{L}^{P}$ are the dispersion force and polar force of the liquid, respectively, mN/m. $\gamma_{s}^{D}$ and $\gamma_{s}^{P}$ can be calculated by the following equations:

(5) $$\gamma_{L1}\left(1\;+\;cos\;\theta_{1}\right)\;=\;2{\left(\gamma_{s}^{D}\gamma_{L1}^{D}\right)}^{1/2}\;+\;2{\left(\gamma_{s}^{P}\gamma_{L1}^{P}\right)}^{1/2}$$

(6) $$\gamma_{L2}\left(1+cos\;\theta_{2}\right)=2{\left(\gamma_{s}^{D}\gamma_{L2}^{D}\right)}^{1/2}+2{\left(\gamma_{s}^{P}\gamma_{L2}^{P}\right)}^{1/2}$$

where $\gamma_{L1}$ is the surface free energy of water, mN/m; $\theta_{1}$ is the contact angle of water on the rock surface, °; $\gamma_{L1}^{D}$ and $\gamma_{L1}^{P}$ are the dispersion force and polar force of the deionized water, respectively, mN/m. $\gamma_{L2}$ is the surface free energy of n-hexadecane, mN/m; $\theta_{1}$ is the contact angle of n-hexadecane on the rock surface, °; $\gamma_{L2}^{D}$ and $\gamma_{L2}^{P}$ are the dispersion force and polar force of n-hexadecane, respectively, mN/m.

### 5.7. Liquid Level Measurements

A glass capillary tube was used to obtain the liquid levels of deionized water and n-hexadecane under the influence of PTQs at room temperature and atmospheric pressure. Before testing, the tube was aged in solutions of different sample concentrations for one week at 60 °C. The relationship between the contact angle and liquid level is given as the follow equation:

(7) 2σcos θ=ρghr

where θ is the contact angle of liquid on the surface of the capillary tube, °; σ is the surface tension of the liquid, mN/m; ρ is the density of the liquid, kg/m^3^; g is the gravitational acceleration, N/kg; h is the liquid level, mm; r is the diameter of the capillary tube, m. It was found that when the contact angle of the liquid medium was less than 90°, the liquid level was greater than 0 mm. A contact angle of 0° corresponds to a liquid level of 0 mm, and the liquid level reduces to under 0 mm when the contact angle is higher than 0°.

### 5.8. Spontaneous Imbibition

A spontaneous imbibition experiment was also used to evaluate the influence of the water-lock-breaking agent on the wettability of rock. Firstly, the original cores were washed by toluene and dried in an oven at 60 °C for 24 h. Secondly, the core was saturated by 2PV of the water-lock-breaking agent using the core-flooding setup. Then, the saturated core was aged in a core holder at 60 °C for 72 h. Thereafter, they were taken out and dried in the oven at 80 °C for 24 h. Thirdly, the original and treated cores were submerged completely in the imbibition brine or n-hexadecane at room temperature with zero initial water saturation. The weight of the imbibed liquid was recorded over time using a scale with an accuracy of 0.0001 g until the weight showed no obvious change. Under the influence of the water-lock-breaking agent, the variations in imbibition weight for brine and n-hexadecane indicate of the gas-wettability alteration of the original rock. 

## 6. Conclusions

In this paper, to eliminate the water lock damage in tight sandstone and reduce the capillary resistance, amphiphilic polymer-modified titanium quantum dots were synthesized via in situ polymerization, showing a hyper-branched structure and synergy with fluorocarbon surfactants to break the water lock effect in tight gas reservoirs. The obtained conclusions can be given as follows:

(1) The synthesized PTQs with a median size of 3.6 nm and significant green fluorescence have zwitterionic hydrophilic groups and the hydrophobic structure of long-chain groups, which can freely migrate throughout the tight reservoir and arrange on the interface of the gas–water phase.
(2) The surface tension significantly decreased from 72 mN/m to 15.2 mN/m under the influence of PTQs, especially in combination with fluorocarbon surfactants. Meanwhile, PTQs exhibited excellent salt- and thermostability due to their specific structure.
(3) The hydrophobicity of sandstone was enhanced by PTQs, but its amphiphobicity of was increased only under the combined effects of PTQs and fluorocarbon surfactants. Hence, the liquid level, surface free energy, and imbibition capacity were powerfully inhibited by this interaction.
(4) The strong surface activity of PTQs results from their specific molecular structure, which enables electrostatic attraction, the quantum size effect, hydrogen bonding, and van der Waals forces between the inter-polar molecules of PTQs and the surface of sandstone, thereby effectively eliminating the water lock effect. Simultaneously, the synergistic effect between PTQs and fluorocarbon surfactants further improves surface tension reduction and wettability alteration.

## Figures and Tables

**Table 1:** Basic properties of sandstone cores.

No.	Diameter (cm)	Length (cm)	Porosity (%)	Permeability (mD)
1#	2.51	5.89	10.32	0.28
2#	2.54	5.62	10.25	0.25

## Data Availability

All data generated or analyzed during this study are included in this published article.
